# Improving the diagnostic yield of echocardiography in acute ischemic stroke: A quality improvement project in a community hospital in Maryland, USA

**DOI:** 10.22088/cjim.14.4.640

**Published:** 2023

**Authors:** Emran A El-Alali, Laith N Maali

**Affiliations:** 1 Department of Internal Medicine, Anne Arundel Medical Center, Annapolis, Maryland, USA; 2 Department of Neurology, University of Kansas Medical Center, Kansas City, Kansas, USA

**Keywords:** Acute ischemic stroke, Cardiac source of embolism, Cost-effectiveness, Echocardiography, Quality improvement.

## Abstract

**Background:**

Echocardiography is routinely ordered in acute ischemic stroke workup. No standardized or structured criteria is used to select or exclude echocardiography in such settings. Moreover, the diagnostic yield of echocardiography in stroke is low in our medical center. This article presents newly proposed selection criteria for echocardiography in ischemic stroke workup.

**Methods:**

A quality improvement project was implemented in a 385-bed community hospital in Maryland, USA. A computerized decision support tool consisting of new criteria for selecting echocardiography in ischemic stroke workup was created. 639 patients hospitalized with ischemic stroke were followed-up over 12 months after intervention, and 686 matched-controls with ischemic stroke were retrospectively analyzed from the 12 months prior to intervention. Cost-effectiveness and diagnostic yield of echocardiography in ischemic stroke were measured before and after intervention.

**Results:**

Following intervention, the diagnostic yield of echocardiography in ischemic stroke workup significantly increased by 51% (from 3.8% to 7.8%, odds ratio (OR) 2.1, *P= 0.01*). The number of echocardiography studies needed to detect and treat one patient with a cardiac source of embolism was reduced from 50 to 25 studies. The overall use of echocardiography in stroke workup significantly decreased (OR 0.4, *p *< 0.001). Patients with lacunar infarcts or atrial fibrillation had significant reduction in echocardiography (OR 0.2, *p *< 0.001 and OR 0.4,* p *< 0.001, respectively).

**Conclusion:**

The new criteria for echocardiography selection in hospitalized patients with ischemic stroke significantly improved the cost-effectiveness and the diagnostic yield of echocardiography and reduced unnecessary echocardiography in lacunar infarcts or atrial fibrillation.

Problem description: Globally, according to the Global Burden of Disease Study, the prevalence of stroke in 2017 was 104.2 million people with 6.2 million deaths were attributable to stroke. For ischemic stroke in particular, the prevalence was 82.4 million, representing a 16.1% increase in the ischemic stroke prevalence rate from 2007 to 2017 (1, 2). In the United States of America, stroke is the leading cause of disability and the fifth leading cause of death. Stroke-related costs in the United States came to nearly $46 billion between 2014 and 2015 (2). Between 2012 and 2030, total direct annual medical stroke-related costs are projected to triple, to $184.1 billion (3, 4). Transthoracic echocardiography accounted for 11% and more than $1.1 billion of total Medicare diagnostic imaging spending in 2010 (5). 

Patients who are hospitalized with acute ischemic stroke (AIS) require neurological and, sometimes, cardiac evaluation. Transthoracic echocardiography (TTE) is often ordered during the evaluation of AIS, either based on the healthcare provider’s clinical judgment for the presence of a cardiac source of embolism (CSE) or routinely as part of comprehensive evaluation of stroke. The clinical judgment for a CSE has not been standardized. No specific criteria are used to determine the probability of CSE, or who would benefit from TTE in AIS.

**Available knowledge**: The diagnostic yield of TTE in ischemic stroke has generally been low. In our medical center, the diagnostic yield and utility of TTE were retrospectively studied in stroke patients who were admitted between 2012 and 2013 and showed only one positive TTE, that was a left ventricular thrombus, as a CSE out of 371 studies done (i.e., a yield of 0.27%) (6). The hospital is a 385-bed community hospital located in Annapolis, Maryland, considered as a stroke-accredited center, and receives 600-800 stroke admissions annually.

Probable reasons for the low yield shown at our medical center were: Only 10 patients were younger than 45 years in the above study, and patent foramen ovale (PFO), isolated atrial septal aneurysm or cardiomyopathy were not included as cardiac sources of embolism.

Bubble studies “saline agitated contrast echocardiography” to detect PFO with shunting were rarely and randomly ordered in stroke patients at our medical center. Many patients younger than age 60 years, who benefit from closure of PFO to prevent recurrent stroke, did not have bubble study done. Results from a larger study at another facility showed even a lower yield (0.2%, 4 of 2464) for TTE in detecting a direct CSE (thrombus, vegetation or mass) (7).

The American Heart Association/ American Stroke Association (AHA/ASA) has considered echocardiography in AIS workup as reasonable (class 2a) rather than recommended (class 1) for the prevention of recurrent stroke (8). In many patients, appropriate evidence-based treatment for secondary prevention of stroke can be selected without the use of echocardiography. Many randomized clinical trials (RCTs) which provided the current best evidence for secondary prevention treatments for AIS did not require TTE for patient selection (9-16).

**Rationale**: In the literature, studies showed that the yield and impact of echocardiography in stroke can increase if proper patient selection is made, (17, 18) and showed that educational efforts and sessions were effective in reducing the utilization of specific diagnostic tests including echocardiography in stroke patients (19).

**Specific aims**: The purpose of this quality improvement (QI) project was to increase the diagnostic yield of echocardiography in detecting cardiac sources of embolism in patients hospitalized with acute ischemic stroke by proper patient selection based on their probability of having a high-risk embolic condition. This was expected to increase the overall cost-effectiveness of AIS workup and reduce low yield or unnecessary imaging studies. Also, the goal was to avoid TTE in patients with AIS that would not make any changes to management and prognosis like in atrial fibrillation, lacunar infarcts, aortic thromboembolism or symptomatic carotid artery disease.

## Methods

**Context**: In our medical center, multiple attempts to reduce the use of unnecessary echocardiography in stroke were made by the cardiology department physicians through instructions and education to the hospital medical staff, but resulted in no significant changes in this setting. No factors were expected to interfere with our results since there were no other interventions or a structured criteria in place to decrease low yield testing in stroke. So, outcomes should solely be the result of our project intervention.

**Intervention**: This is a QI project focused on implementing a structured selection criteria when ordering TTE in patients with AIS. Eligibility included adult patients 18 years and above who were hospitalized between July 2020 and July 2021 with the diagnosis of AIS based on symptoms of stroke, magnetic resonance imaging (MRI) finding of brain infarct, or clinical determination of transient ischemic attack (TIA) (20). The criteria include a list of conditions or situations with high risk for cardiac source of embolism which recommend the utilization of TTE in the workup, and situations where TTE is not recommended given the proven lack of its yield, like in lacunar strokes, or when it does not change management.

The selection criteria are adopted from the guidelines of the American Society of Echocardiography (ASECHO) for the use of echocardiography in the evaluation of a CSE and the Causative Classification System for ischemic stroke (CCS). TTE not recommended in:

1. Lacunar infarcts: Defined as single or few subcortical infarcts in the white matter, basal ganglia, thalamus or pons.
2. Known atrial fibrillation or flutter
3. Extracranial carotid artery disease including significant (>50%) stenosis, occlusion, or dissection ipsilateral to the stroke
4. Suspicion of aortic source of embolic stroke (eg, recent invasive vascular procedure or surgery)

TTE recommended in: (21, 22)

1. Recent acute myocardial infarction
2. Cardiomyopathy with depressed left ventricular function <30%
3. Prosthetic cardiac valve
4. Rheumatic heart disease or severe mitral stenosis
5. Suspected infective endocarditis
6. Brain infarcts in bilateral anterior, or anterior and posterior vascular territories
7. Large cortical infarct
8. Patients younger than 60 years with no traditional stroke risk factors (requires agitated saline study (bubble study) in addition)

The criteria were incorporated into the hospital’s computerized physician order entry (CPOE) after project approval and was applied whenever TTE was ordered in stroke or TIA. Staff education about this project was provided during the stroke committee meetings and the hospitalist huddles. 

**Figure 1:**
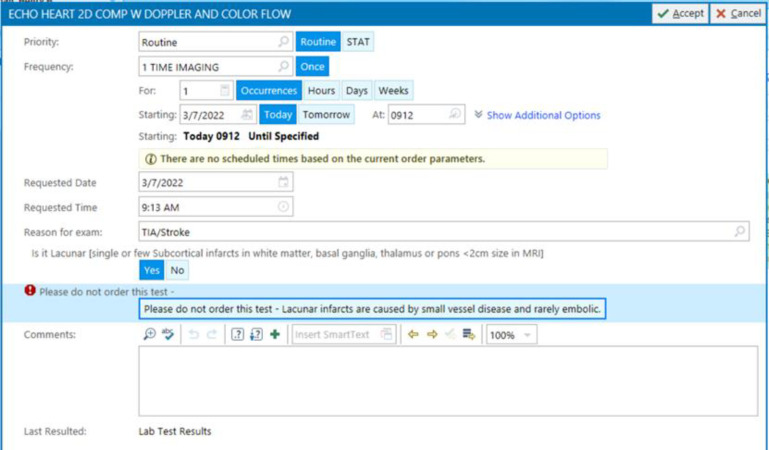
Clinical decision support system to determine the need for echocardiography in ischemic stroke – Step 1

**Figure 2:**
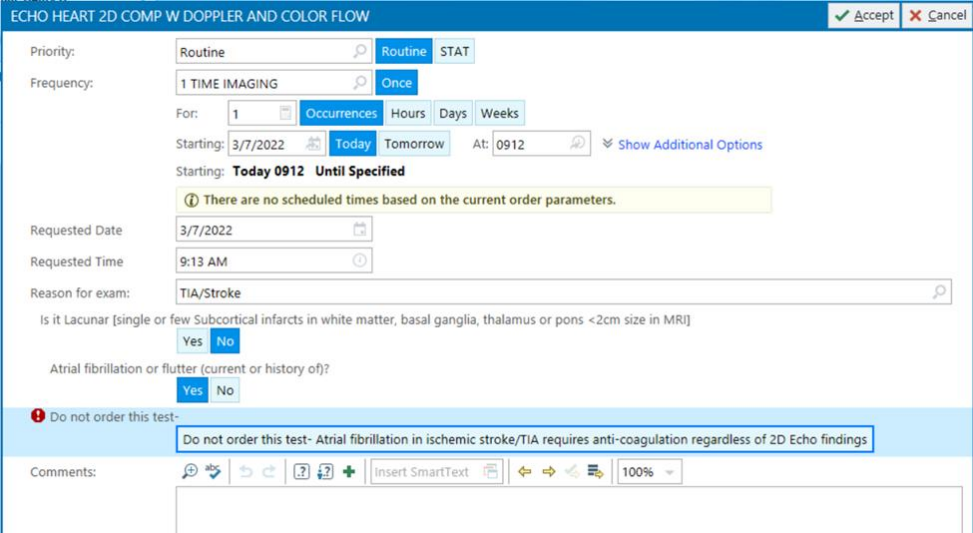
Clinical decision support system to determine the need for echocardiography in ischemic stroke – Step 2

**Figure 3:**
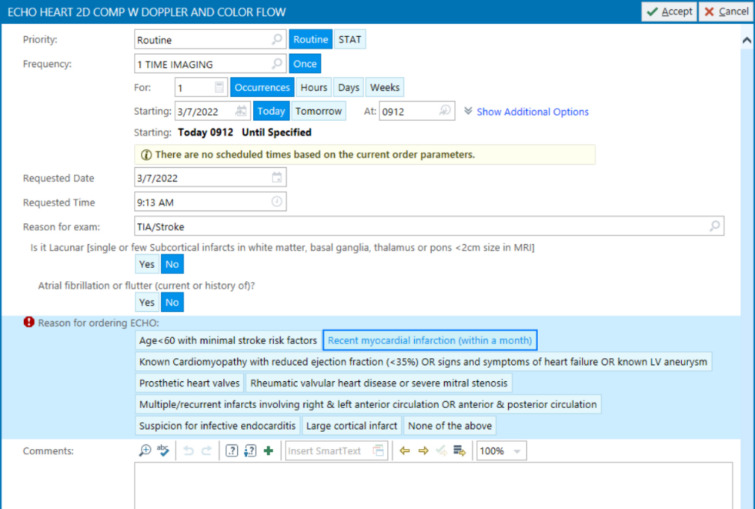
Clinical decision support system to determine the need for echocardiography in ischemic stroke – Step 3

Technically speaking, when the physician orders an echocardiography through the CPOE, a cascade of questions appears. The first general question asks about the reason for the test, and in this case “TIA/stroke” will be chosen from the dropdown list. Then, the second question appears “Is it lacunar stroke?”, if yes is selected, then a message appears “Lacunar infarct is caused by small vessel disease and is rarely embolic”, explaining why the test is not needed in this situation (figure 1). If the answer to the second question “Is it lacunar?” was no, then the third question appears asking “Does patient have atrial fibrillation or flutter?”, if yes is selected, then a message appears “Atrial fibrillation requires anticoagulation regardless of echocardiography findings”, explaining why the test is not needed in this case (figure 2). If the answer to the third question “Does patient have atrial fibrillation or flutter?” was no as well, then echocardiography can be ordered, and the list of “TTE recommended criteria” appears from which the doctor can select the appropriate reason for the test (figure 3), like for example “recent acute myocardial infarction”, or “history of cardiomyopathy”, etc. This way echocardiography will be discouraged in cases of lacunar infarct or atrial fibrillation using the “TTE not recommended” list first, and if none of those present then the test can be ordered. If echocardiography was needed for any different reason other than detecting a cardiac source of embolism in a patient with stroke, then the reason for the test in the first question will be chosen differently, like “heart murmur, or chest pain, etc.” rather than “TIA/stroke”.

The project leader received constant feedback, was responsible for chart auditing and data gathering, and secured all patients' data anonymously in a password-protected software. Physicians were clearly informed about the evidence-based nature of the selection criteria. Radiology department was made aware of this project and the importance of reporting lacunar infarcts as “Lacunar” so it can be easily selected from the “TTE not-recommended” list. A definition of lacunar infarct was added to the list as well. Cardiac monitoring staff was made aware of the importance of detecting atrial fibrillation in stroke patients with prompt reporting of such findings.

**Study of the intervention**: All patients who were hospitalized, or discharged from the hospital, with the diagnosis of AIS or TIA after implementing the interventions of the project were followed-up and charts were reviewed on a monthly basis. ICD codes for AIS and TIA at time of discharge were used to extract charts from the electronic medical records.

Data gathering included:

- The frequency of ordering TTE in AIS.
- The provider who ordered TTE (neurologist versus hospitalist)
- The specific reason selected for ordering TTE (from the selection criteria).
- The frequency of positive TTE, and whether the finding has a high or low embolic potential.
- The frequency of ordering bubble studies in stroke patients younger than 60 years with embolic stroke and no traditional stroke risk factors.
- The impact of positive TTE: whether TTE finding results in changes in management.

The control group comprised patients who were hospitalized, or discharged from the hospital, with the diagnosis of ischemic stroke or TIA based on symptoms of stroke, MRI finding of brain infarct, or clinical determination of TIA, (20) over 12 months prior to the intervention, between July 2019 and June 2020, who had similar demographics and risk factors for stroke compared to the study group patients. ICD codes for AIS and TIA at time of hospital discharge were used to extract charts from the electronic medical records. Data analyzed from the control group included the frequency of ordering TTE, the frequency of positive TTE and the provider who ordered it (neurologist versus hospitalist).

The expected outcomes to the project intervention were to obtain the following changes in the study group (i.e., after intervention) compared to the control group (i.e., before intervention): Decrease in the overall frequency of low yield echocardiography in ischemic stroke workup, particularly in cases of single lacunar infarcts or in atrial fibrillation; increase in the cost-effectiveness and diagnostic yield of echocardiography in detecting high-risk cardiac embolic sources which have impact on management; and increase in the bubble studies among the patient population who benefit from PFO closure for recurrent stroke prevention.

**Measures**: We chose cost-effectiveness to measure outcomes because achieving the same or higher number of positive TTE studies after running fewer TTE studies in AIS workup indicates that we reduced testing without missing a potentially positive finding. It also reflects the validity of the intervention. Positive TTE results are divided into findings with high embolic potential and findings with low/undetermined embolic potential based on the ASECHO guidelines and CCS for ischemic stroke. Table 1 show the TTE findings with high versus low risk of CSE (21, 22).

Management-changing echocardiography findings include left atrial or ventricular thrombus, valvular vegetation, atrial myxoma or PFO with concurrent systemic embolization. QI leader assessed compliance and staff knowledge and gathered feedback on the convenience of the project interventions on a monthly basis.

**Analysis**: An electronic data report of how many stroke patients were admitted to the hospital during the intervention was generated by the project leader from the hospital electronic medical records. Data were stored in Excel software. 

Descriptive data collected from chart reviews were analyzed through descriptive statistics using frequency and percentage on how many TTEs were ordered and percentage of stroke patients who had TTE. Odds ratios were used to compare data obtained from the study group after intervention with data obtained from the control group prior to the intervention, and to check for statistically significant differences between those values. The cost-effectiveness of echocardiography in stroke was measured as the number of studies needed to yield one positive result that changes the management. The diagnostic yield of echocardiography was calculated as the percentage of positive studies out of total studies done.

**Ethical considerations**: To protect confidentiality and privacy of all individuals, data were stored in a password-protected computer. The data collected remained anonymous to protect all human subjects. This project was approved by the Clinical Quality Review Committee of our medical center where the project was intended to be carried out. The committee also reviewed this project proposal and determined that it did not meet the definition of human subjects’ research per 45 CFR 46.102 and was eligible to move forward as a quality improvement initiative. IRBNet ID: (1618111-1).

**Table 1:** Classification of TTE findings

**High embolic potential**	**Low/undetermined embolic potential**
Left atrial thrombus Left ventricular thrombus Mitral stenosis or rheumatic valve disease Prosthetic heart valves Valvular vegetation Chronic myocardial infarction with EF<28% Dilated cardiomyopathy Atrial myxoma Papillary fibroelastoma PFO with concurrent systemic embolization	Mitral annular calcification Patent foramen ovale Atrial septal aneurysm Left ventricular aneurysm without thrombus Left atrial smoke (SEC) without A-fib Congestive heart failure with EF<30% Complex atheroma in aortic arch or ascending aorta Apical akinesia Wall motion abnormalities other than apical akinesia Hypertrophic cardiomyopathy Left ventricular trabeculation/non-compaction

A-fib: Atrial fibrillation; EF: Ejection fraction; PFO: Patent foramen ovale; SEC: Spontaneous echo contrast; TTE: Transthoracic echocardiography.

## Results

The criteria were modified over the 12-month period of implementation one item was added to the “TTE recommended” list: “Large cortical infarct”. Two items were added to the “TTE not-recommended” list as well: “The presence of significant carotid artery disease ipsilateral to the infarct” and “the possibility of aortic source of embolic stroke”. Over the course of 12 months after the intervention, 639 patients with acute ischemic stroke were followed-up. The matched-control group consisted of 686 stroke patients over a period of 12 months prior to the intervention.

The frequency of TTE in stroke (i.e., TTE studies per 100 cases of stroke) has significantly declined from 68.6% among the controls, prior to the intervention, to 47.6% in the study group after the project intervention (odds ratio (OR) 0.4, 95% CI 0.3 to 0.5; *p *< 0.001). There was a significant reduction in echocardiography in patients with lacunar stroke after the intervention (OR 0.2, 95% CI 0.1 to 0.3; *p *< 0.001) and in patients with atrial fibrillation and stroke (OR 0.4, 95% CI 0.3 to 0.7;* p *< 0.001). The percentage of positive TTE results showing high embolic-potential findings has significantly increased after the intervention by 51% (from 3.8% to 7.8%. OR 2.1, 95% CI 1.1 to 3.9; *P = 0.01*). Cost-effectiveness of echocardiography in stroke has increased; the number of TTEs needed to detect and treat one patient with a cardiac source of embolism decreased from 50 studies prior to intervention to 25 following the intervention. The frequency of bubble studies, for the detection of PFO with shunting, out of the total cases of stroke has significantly increased from 8% prior to intervention to 13.5% following the intervention (OR 1.8, 95% CI 1.1 to 2.8; *P = 0.02*).

Prior to the project intervention, 77% of TTE studies done in stroke were ordered by hospitalists and 15% by neurologists. Following the intervention, the percentage of TTEs ordered by hospitalists decreased to 63% whereas the percentage of TTEs ordered by the neurologists increased to 22%.Table 2 summarizes the measures before and after the intervention.  Data related to echocardiography in patients with ischemic stroke secondary to aortic thromboembolism or symptomatic carotid artery disease were incomplete after 12 months of this project, because those items were added later on in the intervention.

**Table 2:** Summary of the measures before and after the intervention

	**Before the intervention** **(Control group)**	**Following the** **Intervention (Study group)** **(Study group)**
Total cases of stroke	686	639
Echocardiography studies	497	332
Stroke cases who had echocardiography	68.6% (471/686)	47.5% (304/639) OR 0.4, *P *< 0.001
Echocardiography studies to yield one positive result ^a^	26	13
Echocardiography studies to change management in one case	50	25
Diagnostic yield of echocardiography ^b^	3.8% (19/497)	7.8% (26/332) OR 2.1, *P *= 0.01
Lacunar strokes who had echocardiography	62.8% (135/215)	30.8% (74/240) OR 0.2, *P *< 0.001
Atrial fibrillation with stroke who had echocardiography	63.5% (87/137)	34% (64/173) OR 0.4, *P *< 0.001
Bubble studies	8% (38/471)	13.5% (31/304) OR 1.8, *P = 0.02*
Parentage of TTE ordered by hospitalists vs neurologists	77% vs. 15%	63% vs. 22%

a) High embolic potential finding. b) Percentage of positive echocardiography studies per 100 studies. OR: Odds ratio; TTE: Transthoracic echocardiography.

## Discussion

In our 12-month experience, our QI project resulted in a significant improvement in the diagnostic yield of TTE for detecting high embolic-potential conditions, a significant increase in the bubble studies in those who benefit from PFO closure for secondary stroke prevention, and a decrease in the number of TTE studies needed to make a change in the management in one case.

To our knowledge, this is the first study to establish structured criteria for TTE selection or exclusion in patients hospitalized with acute ischemic stroke. It proved its success so far. The criteria represent a computerized clinical decision support system (CDSS) that can help busy providers make the appropriate and up-to-date decision. This QI project has many factors to increase its likelihood of sustainability. Neurologists, cardiologists, and hospitalists recognized the need for standard and structured criteria to select patients for TTE in stroke, and the great majority of healthcare providers were interested in reducing the unnecessary workup. Providers found it convenient to use a CDSS with a predesigned checklist when ordering a test.

**Interpretation**: The diagnostic yield of echocardiography in ischemic stroke increased as a result of selecting the appropriate patients to undergo TTE. The cost-effectiveness of echocardiography in ischemic stroke increased as a result of excluding patients with low probability of a cardiac source of embolism or patients in whom TTE would make no change in management. The percentage of TTEs ordered by neurologists increased following the intervention, because they were more comfortable using the intervention criteria in general as compared to hospitalists. This reflects the higher level of experience among neurologists in stroke management and in gathering more data, particularly data needed to select a reason for TTE from the “TTE recommended” list. Since the intervention is considered a CDSS, our positive outcomes are in agreement with the literature which showed that CDSSs, that link the medical record to appropriateness criteria, have been shown to be effective in reducing unnecessary ordering of tests in a variety of clinical settings (23-25).

**Limitations**: Our project has a few limitations. TTE is a non-invasive study that many healthcare facilities find as a quick and useful tool to rule out a cardiac source of embolism in stroke patients, and such testing may not have a significant financial impact on institutions. Fear of medico-legal consequences (i.e., missing a potentially positive finding in TTE) may hinder the widespread use and compliance to this project.

Knowledge about the presence of atrial fibrillation may not always be available at the time of hospital admission in some patients. Radiologists may not always point to the exact nature and type of the infarct like in lacunar infarcts and may use the term lacunar infarct to describe small infarcts affecting multiple vascular territories which can potentially be embolic.

The new structured criteria for echocardiography selection or exclusion in patients hospitalized with acute ischemic stroke were successful in: Improving the cost-effectiveness and diagnostic yield of echocardiography in detecting high-risk cardiac sources of embolism; reducing unnecessary echocardiography in lacunar infarcts, which are rarely embolic, and in atrial fibrillation, where it does not influence the management; and in increasing the bubble studies to detect PFO with shunting in patients who benefit from PFO closure for secondary stroke prevention. This project is useful as another step in reducing stroke-related cost and expenses, so we expect this project will have sustainability. Using similar CDSSs, we can reduce other unnecessary tests in stroke and other conditions by applying evidence-based criteria to guide the selection of any test or procedure. Next steps should include further follow-up of the intervention results to know the long-term impact and outcomes of reducing low yield echocardiography in stroke workup as a result of this project, particularly on the overall stroke morbidity, length of hospital stay, and on the institution’s workflow.
